# Systematic review and meta-analysis of the effect of bone marrow-derived cell therapies on hind limb perfusion

**DOI:** 10.1242/dmm.050632

**Published:** 2024-05-24

**Authors:** Femke Christina Ching-Chuan van Rhijn-Brouwer, Kimberley Elaine Wever, Romy Kiffen, Jon-Ruben van Rhijn, Hendrik Gremmels, Joost Ougust Fledderus, Robin Wilhelmus Maria Vernooij, Marianne Christina Verhaar

**Affiliations:** ^1^ Department of Nephrology and Hypertension, Regenerative Medicine Center Utrecht, University Medical Center Utrecht, 3584 CX Utrecht, The Netherlands; ^2^ Department of Anaesthesiology, Pain and Palliative Medicine, Radboud University Medical Center, 6525 GA Nijmegen, The Netherlands; ^3^ Institute of Life Sciences and Chemistry, HU University of Applied Sciences Utrecht, 3584 CS Utrecht, The Netherlands; ^4^ Department of Medical Microbiology, University Medical Center Utrecht, 3584 CX Utrecht, The Netherlands; ^5^ Department of Nephrology and Hypertension, University Medical Center Utrecht, 3584 CX Utrecht, The Netherlands; ^6^ Julius Center for Health Sciences and Primary Care, University Medical Center Utrecht, Utrecht University, 3584 CX Utrecht, The Netherlands

**Keywords:** Cell therapy, Hind limb ischemia, Meta-analysis, Perfusion, Systematic review

## Abstract

Preclinical and clinical studies on the administration of bone marrow-derived cells to restore perfusion show conflicting results. We conducted a systematic review and meta-analysis on preclinical studies to assess the efficacy of bone marrow-derived cells in the hind limb ischemia model and identify possible determinants of therapeutic efficacy. *In vivo* animal studies were identified using a systematic search in PubMed and EMBASE on 10 January 2022. 85 studies were included for systematic review and meta-analysis. Study characteristics and outcome data on relative perfusion were extracted. The pooled mean difference was estimated using a random effects model. Risk of bias was assessed for all included studies. We found a significant increase in perfusion in the affected limb after administration of bone marrow-derived cells compared to that in the control groups. However, there was a high heterogeneity between studies, which could not be explained. There was a high degree of incomplete reporting across studies. We therefore conclude that the current quality of preclinical research is insufficient (low certainty level as per GRADE assessment) to identify specific factors that might improve human clinical trials.

## Teaser

**Summary:** Studies on bone marrow cell administration in animal models of hind limb ischemia demonstrate improved limb perfusion. Meta-analysis of these studies indicates that the certainty of the evidence is low, limiting applicability to clinical practice.

## INTRODUCTION

Chronic limb-threatening ischemia (CLTI) is the advanced stage of peripheral arterial disease (PAD), which is caused by arterial obstruction. Around 200 million adults worldwide have PAD (Fowkes et al., 2013), of whom approximately 10% develop CLTI (Nehler et al., 2014). Current treatments include surgical revascularization or angioplasty to restore blood flow; however, success rates are low and these treatments do not increase long-term survival. Moreover, many patients are not eligible for the currently available procedures due to surgical risk or comorbidities (Uccioli et al., 2018). The lack of successful long-term treatment options results in severe impact on the quality of life (de Nigris et al., 2007a; Al-Rifai et al., 2019; Haghighat et al., 2019) and a mortality risk of 20% in the first 6 months after initial diagnosis, which increases to 50% over 5 years.

Impaired neovascularization has been implicated as a key pathophysiological feature in CLTI (de Nigris et al., 2007a; Al-Rifai et al., 2019; see Haghighat et al., 2019 for a review). Therapies that improve neovascularization might be a viable option when revascularization is not possible. One potential strategy to augment the neovascularization is to administer bone marrow (BM)-derived mononuclear cells (BM-MNCs) or BM-derived mesenchymal stem cells (BM-MSCs). BM-MNCs are a mixture of different cell types present in the BM, including monocytes, macrophages, pericytes and mesenchymal stem cells (MSCs). These cells have been reported to promote neovascularization and angiogenesis and restore perfusion in ischemic areas by secreting growth factors and cytokines (Yusoff et al., 2019). BM-MSCs are a cell population defined by their ability to differentiate *ex vivo* into cells of various tissues upon stimulation. MSCs also secrete a wide variety of growth factors, cytokines and extracellular vesicles that promote angiogenesis, prevent apoptosis and can modulate immunological responses (Cunningham et al., 2018). Both BM-MNCs and BM-MSCs have been proposed as highly promising candidates for therapeutic intervention in CLTI.

Prior to the initiation of clinical trials, the efficacy of BM-derived cells in CLTI was assessed in relatively few preclinical trials, primarily using animal models for hind limb ischemia (HLI). Transplantation of these cells resulted in increased tissue perfusion, increased angiogenesis and reduced limb loss (Yoshida et al., 2003), as reviewed in Sprengers et al. (2008). At the same time, clinical pilot studies in patients with CLTI were conducted, which suggested translational success from preclinical models. However, these clinical studies were often small, poorly controlled and not masked (Peeters Weem et al., 2015). This, combined with the short time between development of the first animal models of angiogenesis in 1998 (Couffinhal et al., 1998) and the first clinical trials on limb ischemia in 2002 (Tateishi-Yuyama et al., 2002), raises concerns for translational failure. Indeed, both a larger randomized clinical trial and meta-analyses concluded that there was no significant improvement in major amputation rate, mortality rate or quality of life (Peeters Weem et al., 2015; Teraa et al., 2015, 2013).

Here, we collected and analyzed the preclinical evidence for BM-derived cell interventions. We also critically assessed potential sources of bias and treatment-specific factors (such as dose and administration route) that could influence outcomes to inform future clinical trials.

## RESULTS

### Screening and inclusion strategy

**Fig. 1.**
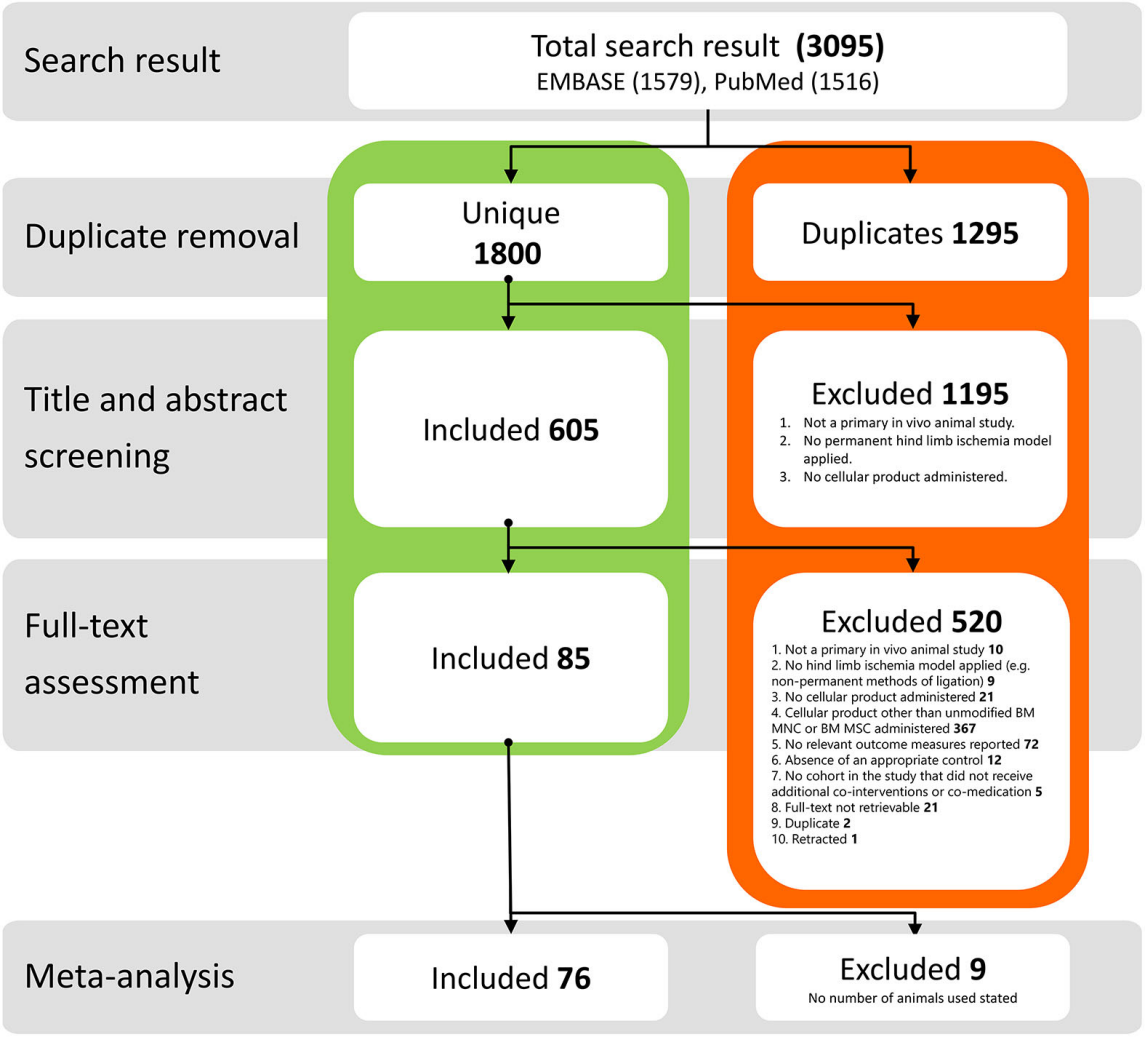
**Flowchart of the search and inclusion strategy.** BM-MNC, bone marrow-derived mononuclear cell; BM-MSC, bone marrow-derived mesenchymal stem cell.

The search strategy resulted in 3095 articles from PubMed and EMBASE. After removal of duplicates, title and abstract screening, and assessment of the full text, 85 studies were included in the systematic review (Fig. 1). Studies excluded at the full-text assessment stage and the reasons for exclusion can be found in Table S1.

### Study characteristics

Table 1 presents an overview of study characteristics. Most studies were performed in mice (81%), 13 (15%) studies were performed in rats and three (4%) in rabbits. 38% of the studies did not report the sex of the animals, 53% reported using male animals, 13% reported using female animals and one study used both sexes. In 92% of studies, animals without comorbidities were used. Other studies used (multiple) animal models with comorbidities: four studies used animal models of diabetes, two used obese animals, four used animals with atherosclerosis and one study used animals with hypertension. In 35% of the studies, the animals were immunocompromised, which correlated with the administration of xenogeneic cells. The method of inducting HLI varied and ranged from ligation or electrocautery at a single point to full excision of the artery (Table S2).

**Table 1.**
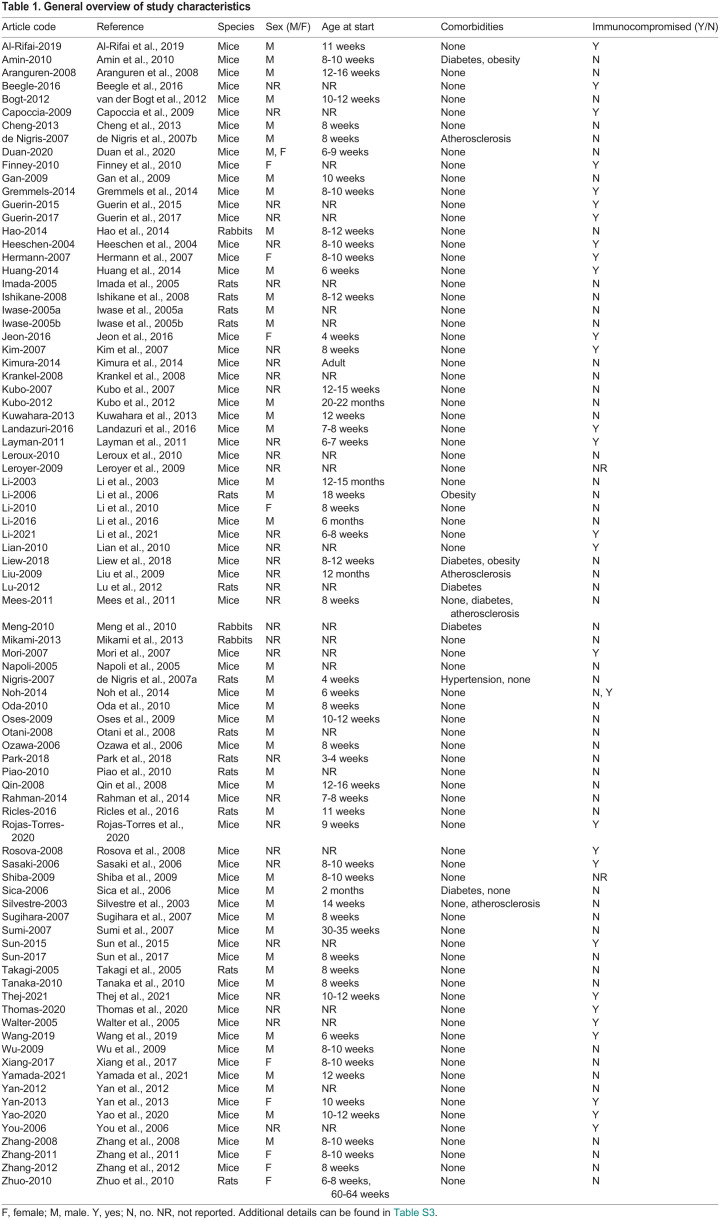
General overview of study characteristics

Of the three cell models used, BM-MSCs were used in 53% of studies, 28% used BM-MNCs and 21% used BM cells. One study compared BM cells and BM-MSCs versus the control group, which leads to a total of >100% in the summation of studies. Multiple administration methods were described, of which the majority comprised intramuscular (74%) or intravenous (21%) injection, and two studies compared intramuscular versus intravenous administration. Two studies used intra-arterial administration (Li et al., 2021; Liu et al., 2009) and two studies injected the cells intracardially (Finney et al., 2010; Rosova et al., 2008). Finally, there was a single study that injected intraperitoneally (Noh et al., 2014). The animals were administered cells at a single time point in 97% of the studies. However, 57% used multiple injection sites. The doses varied between 5×10^4^ cells and 2.5×10^7^ cells, with a median of 1×10^6^ cells. The median dose per injection was 1×10^5^ cells, with a range of 1.7×10^4^ to 2.5×10^7^ cells. The median timepoint of intervention was 24 h after HLI induction, with a range of −24 to 336 h. The follow up ranged from 7 to 70 days, with a median of 21 days. Most studies assessed the perfusion at multiple time points (72%).

The cells used for treatment were mainly allogeneic (60%), 31% were xenogenic and 9% were autologous. A single study compared both xenogenic and allogenic origins. In the case of xenogenic cells, the majority were human cells. Five studies included cells from a diseased donor (either human or a disease model). Most studies did not report the sex of the donor animals (61%) and most (85%) did not provide information on whether the cells were cryopreserved. The remaining 15% reported cryopreservation.

### Risk of bias assessment

**Fig. 2.**
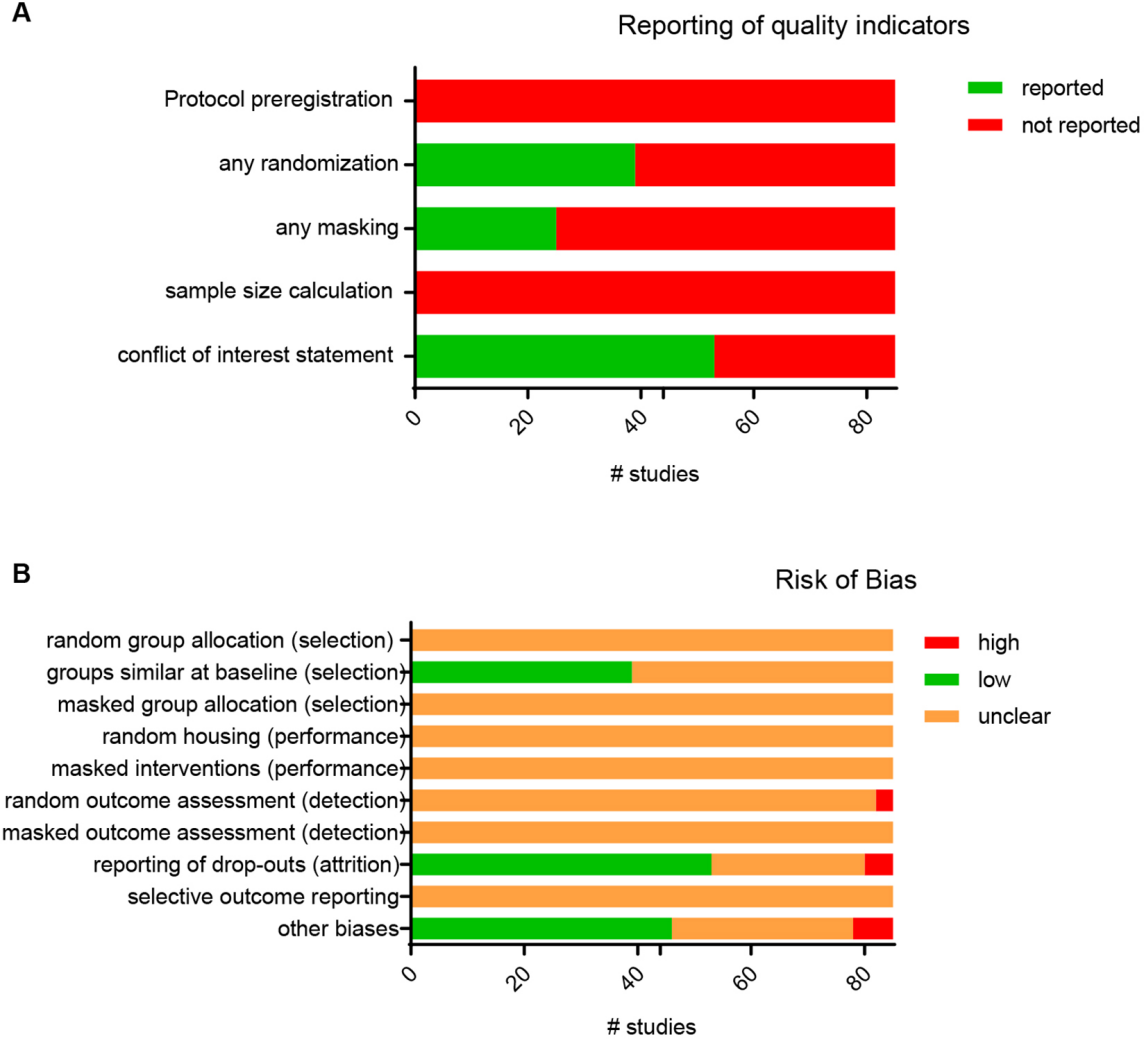
**Reporting of study quality indicators and risk of bias assessment.** (A) Number of studies reporting specific quality indicators. (B) Risk of bias assessment using the SYRCLE risk of bias tool. Each item was scored as having a low, high or unclear risk of bias. *n*=85 studies.

Many studies showed a lack of clear reporting in the risk of bias items, which results in an ‘unclear’ score on most of the bias items. Randomization and/or masking was mentioned in 46% and 29% of studies, respectively. However, studies only described randomization of *ex vivo* or *in vitro* samples during analysis, not of the actual treatment. The randomization process that was used to allocate treatment to animals or the method by which samples were masked was never clearly reported, and none of the studies reported sample size calculation or preregistration (Fig. 2A). Half of the studies (48%) clearly reported experimental groups with similar baseline characteristics at the start of the experiment (Fig. 2B). The number of animals included in different experiments was often (68%) not clearly mentioned, which resulted in an increased risk of attrition bias (Fig. 2B). A full overview of scores for each study can be found in Table S4.

### Meta-analysis

#### Overall effect

**Fig. 3.**
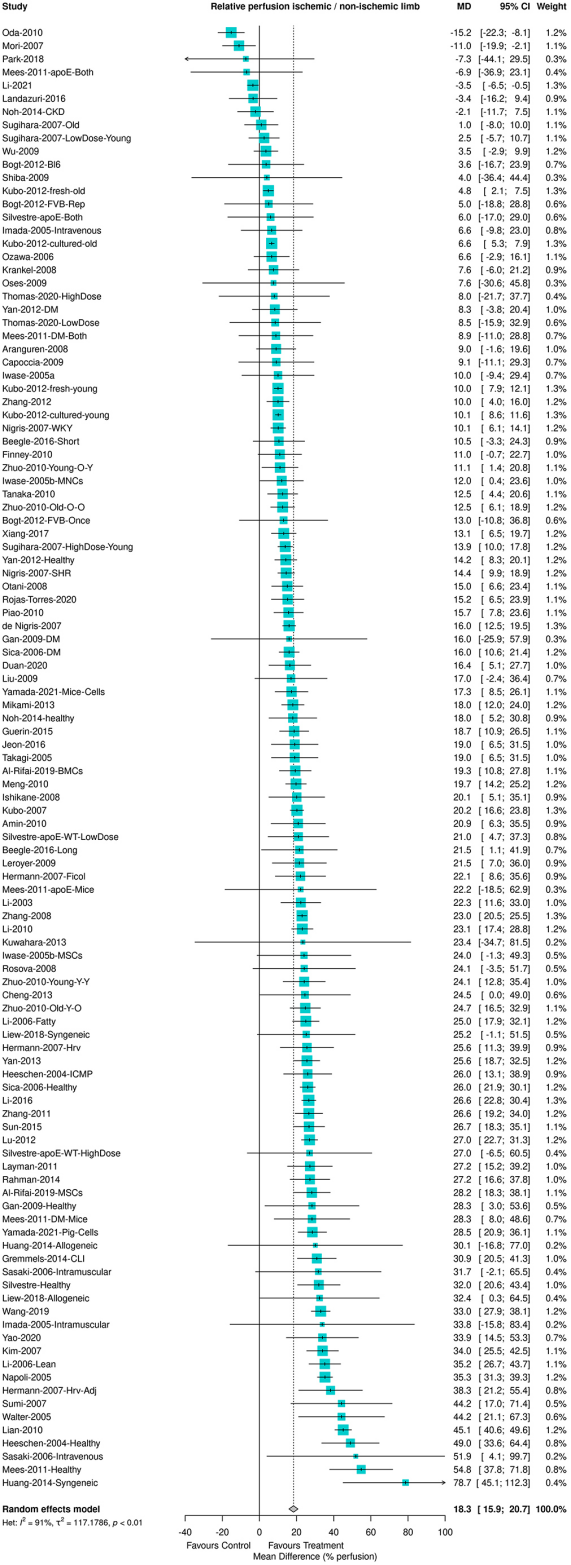
**Forest plot showing an increase in the relative maximum perfusion between the ischemic and the non-ischemic limb after treatment with BM-MNCs or BM-MSCs in animal models of hind limb ischemia.** Effects were plotted as the mean difference (MD) and pooled using a random-effects model. The diamond represents the estimate of the mean difference across all studies and the dashed line represents the point estimate of the mean difference. *n*=111 comparisons from 76 studies. BM-MNC, bone marrow-derived mononuclear cell; BM-MSC, bone marrow-derived mesenchymal stem cell.

Nine studies included in this review did not report the number of animals used and were excluded from the meta-analysis. The remaining 76 studies included 111 individual comparisons. The effect of BM cell treatment on limb perfusion was assessed using 1053 animals in total. Meta-analysis showed an overall effect size of 18.3 [95% confidence intervals (CI)=15.9-20.7, *P*<0.001], suggesting that BM cell treatment results in better perfusion compared to that of the control condition (Fig. 3). However, the heterogeneity between studies was very high at 91%.

#### Subgroup analysis and exploring heterogeneity

With the aim of identifying determinants of treatment efficacy, subgroup analyses were performed using meta-regression on ten variables that were previously identified in the literature as potentially contributing to efficacy (Table S5). After correcting for multiple testing, none of the variables significantly affected the standard mean difference, nor did they explain heterogeneity, and the residual I^2^ (indicating the remaining variation due to between-study heterogeneity) remained high in all analyses (Table 2). We were thus unable to identify the source of the high heterogeneity. Forest plots for each subgroup analysis can be found in Figs S1-S9.

**Table 2.**
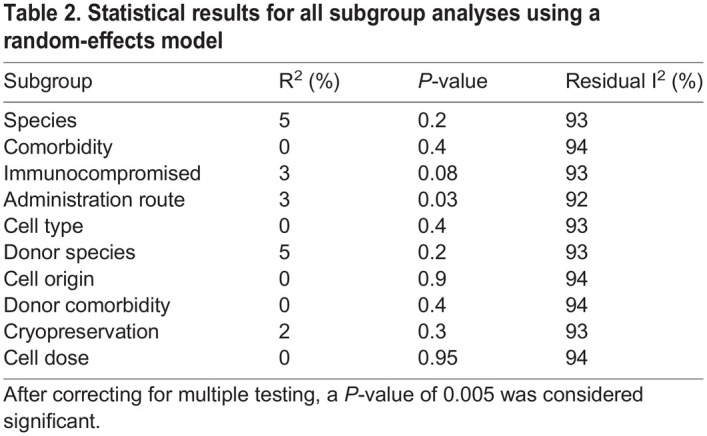
Statistical results for all subgroup analyses using a random-effects model

**Fig. 4.**
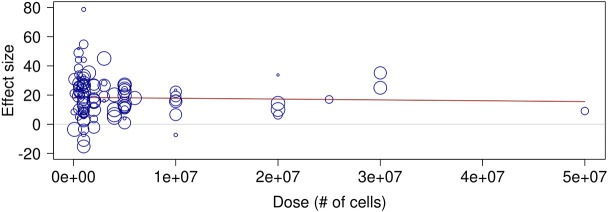
**Meta-regression analysis of effect size versus dose (number of cells).** The red line represents the trend of the effect size. *n*=111 comparisons from 76 studies.

Our previous work suggested a trend towards less efficacy at a higher dose (Gremmels et al., 2014). We thus assessed the effect of the dose on limb perfusion. Meta-regression shows an almost flat line and thus no correlation between the effect size and the dose (Fig. 4). As such, the dose does not explain the heterogeneity between studies, showing a R^2^ of 0.00% and a *P*-value of 0.9.

#### Sensitivity analysis

**Fig. 5.**
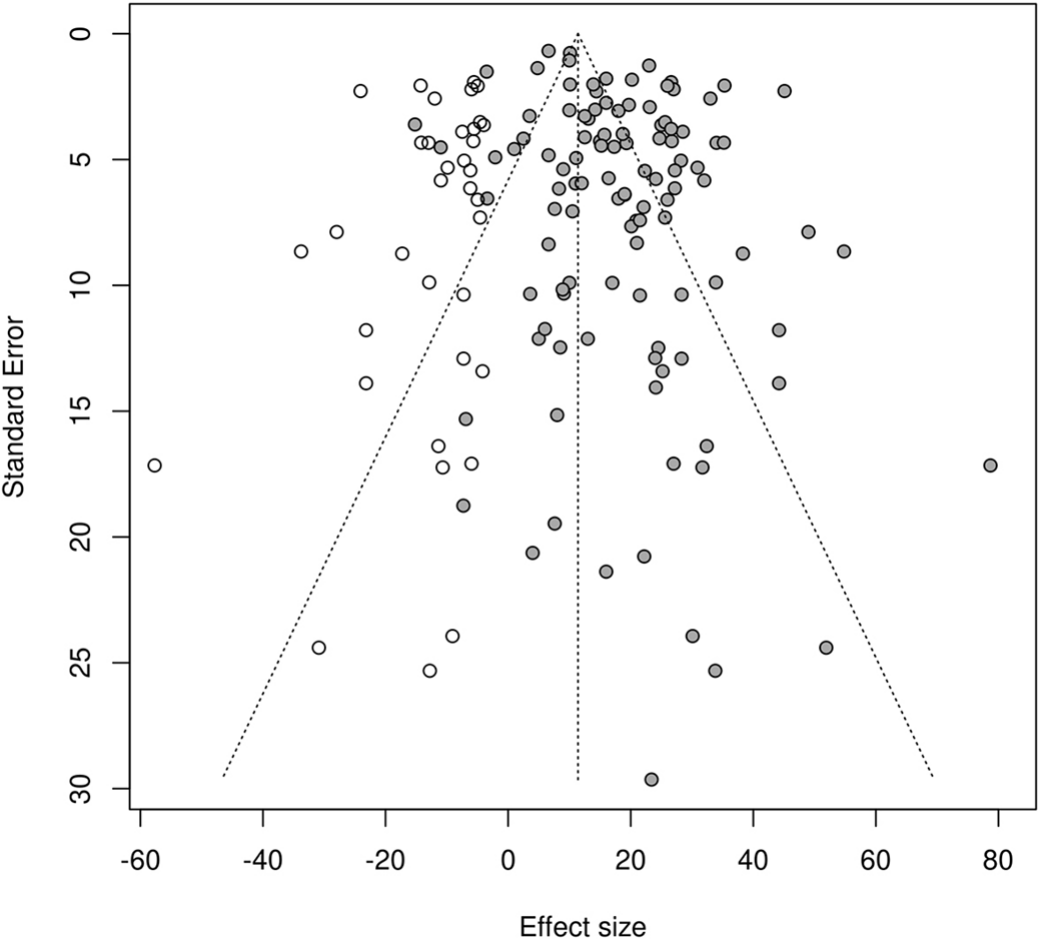
**Publication bias assessment using trim-and-fill analysis added 36 missing studies (open circles) to the original 111 comparisons (filled circles).** The dashed vertical line indicates the pooled estimate based on the model. A pseudo confidence interval region is drawn around this value with bounds equal to ±1.96×s.e.

A sensitivity analysis was performed using the perfusion measured at the latest time point for each study as opposed to the time point of maximum perfusion. The overall analysis as well as all subgroup analyses were rerun. The analysis showed an overall effect size of 18.56 (95% CI=14.30-22.81) (Fig. 5), which was similar to the previously observed effect size. The effect, although slightly lower, is still significant. The heterogeneity remains unchanged, and here too, no subgroup analysis showed any significant result.

### Publication bias

The trim-and-fill analysis added 36 studies on the left-hand side of the plot (Fig. 5), which suggests that nearly 25% of datapoints are missing. The pooled effect size was significantly reduced from 18.3 (95% CI=5.9-20.7) to 11.4 (95% CI=8.5-14.3), indicative of a substantial risk of publication bias in our dataset.

## DISCUSSION

In this systematic review and meta-analysis, we show that BM cell therapy ameliorates perfusion deficits in the preclinical HLI model. Despite marked differences between approaches, such as dose, administration route, animal age and comorbidities, BM cell therapy was beneficial in all cases.

The failure of cell therapy clinical trials in CLTI resulted in extensive discussion of possible contributing factors in the literature. The clinical literature suggested geographic and gender-related factors (Teraa et al., 2013), as well as the *ex vivo* manipulation, including cryopreservation, of the cells. In our synthesis of the preclinical evidence, we assessed several additional factors that could have affected therapeutic efficacy. These are: species, comorbidity, presence of immunodeficiency, administration route, cell type, cell origin and donor characteristics, cryopreservation, and cell dose.

Synthesis of the evidence indicates severe limitations in the external validity of preclinical CLTI models: CLTI is more prevalent in women than in men (Sigvant et al., 2007; Kavurma et al., 2023), but in preclinical trials, male animals are overrepresented; only 13% of the studies included female animals. Animals are generally healthy young adults, whereas patients with PAD are often elderly and have comorbidities such as diabetes or atherosclerosis. Only 11 studies included in this review used animals with comorbidities. Lastly, there was often only a short delay between HLI induction and treatment. Although the timing of the treatment is similar to the timing of treatment in acute peripheral ischemia, CLTI generally has a more chronic course (Londero et al., 2014). It has been well established that the local tissue environment is markedly different in chronic ischemia compared to that in an acute ischemic setting, which reduces the external validity of the treatment setup used in most HLI studies.

Our meta-analysis shows that in the HLI model, BM cell treatment has an overall beneficial effect, resulting in an increase in perfusion of the affected limb. However, these findings need to be interpreted with caution as there was considerable heterogeneity. Unexplained high heterogeneity suggests a shortcoming in our understanding of what drives the differences between CLTI models and the efficacy of stem cell treatment therein, making it questionable as to whether these models can be used to inform research in humans. We conducted subgroup analyses to explore possible factors that contribute to treatment efficacy; however, none of them showed a significant difference or lowered heterogeneity.

Most of the studies were conducted in mice, but none provided a rationale for this choice. Practical considerations may have played a role, as mice are cheap both to acquire and keep, and a wide variety of transgenic mice is available for purchase, especially in comparison to rats or larger mammals. However, genetic disease models were only rarely used. Although studies in cardiovascular disease have shown that large animal models show increased external validity and considerably greater similarity to humans (Tsang et al., 2016), these were completely lacking in our dataset. Lastly, several studies suggest that the specific background (strain) of model animals influenced their recovery after HLI. However, this mostly concerned immunocompromised mice (Helisch et al., 2006; Chalothorn and Faber, 2010), and our subgroup analysis did not reveal an effect of immunostatus.

Cryopreservation did not affect perfusion. In human clinical trials that failed to meet endpoints, cryopreservation and thawing protocols have been mentioned as possible contributing factors (Xu et al., 2012; Galipeau, 2013; Francois et al., 2012). Additionally, cryopreservation can affect *in vitro* efficacy (Bahsoun et al., 2019). Our analysis suggests that cryopreservation does not affect BM-derived cell therapy in the context of perfusion, although the high heterogeneity in our cohort precludes firm conclusions.

Our findings are similar to human clinical meta-analyses, which reported increased perfusion after cell-based treatment in placebo-controlled trials (Peeters Weem et al., 2015; Bahsoun et al., 2019). However, in human clinical studies, cell-based treatments neither reduced amputation rates nor increased survival rates (Peeters Weem et al., 2015). These outcomes are only very rarely reported in the animal studies assessed in this review, and a meta-analysis using these outcomes would not be possible.

Due to incomplete reporting, it is difficult to assess the rigor and quality of the reported data extracted in this review, leaving all studies at an unclear risk of several types of bias. For example, descriptions of randomization and masking methods were often not provided, and selection bias on the reported data could often not fully be ruled out. Importantly, none of the studies reported an *a priori* power calculation, which puts the results at risk of reporting spurious findings, outcome switching and hypothesizing after results are known (HARKing). These issues decrease our confidence in the evidence base. Improved reporting of both study characteristics as well as outcome validation is an essential next step towards increasing quality of animal research. High risk of bias has been suggested as a possible reason for the initial promising results in human clinical trials, with later randomized controlled trials showing no benefit of cell-based treatment over the placebo (Rigato et al., 2017). It is possible that similar factors play a role in preclinical studies; however, the lack of clear reporting in most studies precludes meaningful analysis.

Our publication bias assessment using trim-and-fill analysis suggests that nearly 25% of the evidence in this field remains unpublished, indicating an overestimation of the treatment effect, although a significant effect remains after correcting for missing studies. Due to the high heterogeneity and the limited variation in sample size between the studies, this has to be interpreted with care. In view of the general prevalence of publication bias in animal research, we encourage researchers to make use of preregistration, registered reports and data repositories to make all animal data available, regardless of their significance level.

### GRADEing the certainty in the evidence

Based on the study characteristics, risk of bias assessment and meta-analysis results, we performed a ‘Grading of Recommendations, Assessment, Development and Evaluations’ (GRADE) assessment in line with the recommendations for animal studies (Aref et al., 2019). The certainty in the evidence was downgraded due to risks of bias (serious), inconsistency (very serious), indirectness (serious) and publication bias (serious) (Table 3). A dose-response effect was absent, and although there was consistency across rodents, we deem the variety in species included too limited to upgrade the certainty in the evidence. Overall, the certainty in the evidence was classified as low.

**Table 3.**
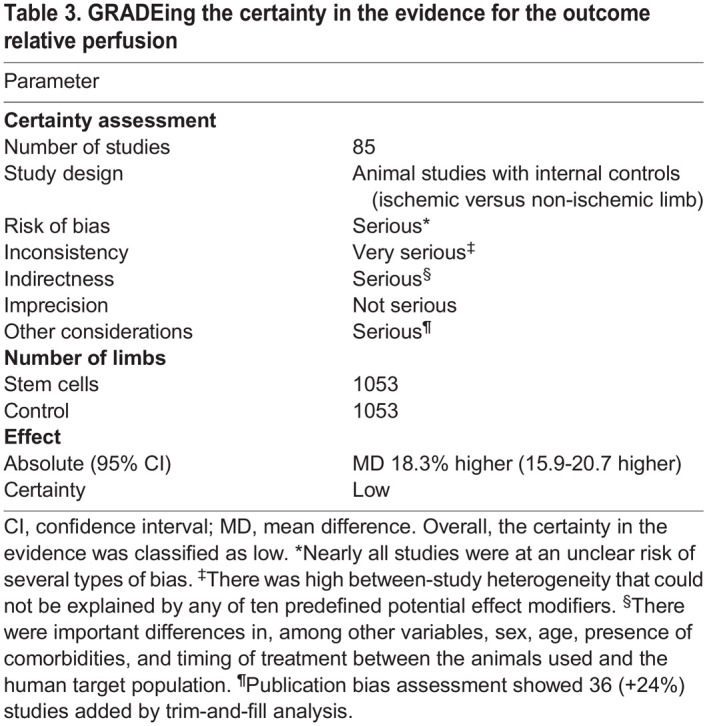
GRADEing the certainty in the evidence for the outcome relative perfusion

### Strengths

This review provides a comprehensive overview of animal studies investigating the use of BM-derived stem cells in HLI. The meta-analysis was conducted using rigorous and robust methods. The protocol was preregistered (van Rhijn-Brouwer et al., 2021) and peer reviewed (van Rhijn-Brouwer et al., 2021).

### Limitations

As we selected relative perfusion as primary outcome measure, this resulted in the exclusion of several early preclinical studies that used histological parameters as outcome measures, including most studies that served as a foundation for the first clinical trials. Relative perfusion represents a functional measure and is therefore viewed as more clinically relevant compared to histology. As relative perfusion is normalized to the contralateral control limb, we also expected this to minimize heterogeneity.

Additionally, in the included studies, a range of methods to induce HLI was used. It has previously been suggested that some methods used to create HLI affect perfusion to different degrees (Aref et al., 2019). We did not take the specific surgical methods to induce HLI into account in our meta-analysis. Future research should address this potentially important model-related factor.

### Conclusion

Our meta-analysis consistently shows a positive effect of BM-derived stem cells on limb perfusion, although with a high heterogeneity, for which no explanation could be found. An unclear risk of bias and limitations in external validity of the models used might contribute to limited translational success. We were unable to identify specific factors that might affect treatment efficacy. Future animal studies should aim to eliminate the possible causes of the heterogeneity in the dataset by increased adherence to reporting standards and increased quality of study design.

## MATERIALS AND METHODS

This systematic review and meta-analysis was conducted according to a prospectively registered (CRD42021226592) and published (van Rhijn-Brouwer et al., 2021) protocol, and adheres to the 2020 updated PRIMSA reporting guidelines (Page et al., 2021). One amendment to the protocol was made (specification of a sensitivity analysis) as recorded in PROSPERO (https://www.crd.york.ac.uk/prospero/display_record.php?RecordID=226592).

### Search strategy

*In vivo* animal studies investigating the effects of BM-MSCs or BM-MNCs on perfusion after HLI were identified using a systemic search in PubMed and EMBASE via Ovid (https://oce.ovid.com/). Both databases were searched from inception up to 10 January 2022. The search was based on the components ‘ischemia’, ‘limb’, ‘PAD’ and ‘stem cells’ (see supplementary Materials and Methods for the full search strings). Laboratory animal search filters were used to specifically identify animal studies (Hooijmans et al., 2010; de Vries et al., 2014).

### Screening and study selection

After removal of bibliographic duplicates, the retrieved records were screened for eligibility based on their title and abstract. Using our predefined exclusion criteria, records were excluded if they (1) did not present unique outcome data from an *in vivo* animal experiment, (2) did not report on a permanent HLI model or (3) did not involve the administration of a cell product. Subsequently, eligible studies were screened for final inclusion based on their full text. In this phase, only studies investigating BM-MNCs and BM-MSCs were selected for final inclusion. In addition to the criteria above, studies were excluded if (4) the origin of the MSCs was unclear or not specified, (5) the cells were modified after isolation, (6) the animals underwent co-interventions or received co-medications, (7) no appropriate control group was present or (8) our primary outcome, the perfusion of the ischemic limb relative to that of the control limb, was not reported. No language restrictions were applied. In both screening phases, each record was assessed by two independent reviewers who were not aware of each other's screening decisions. Disagreements were resolved through discussion or, if consensus could not be reached, by a third reviewer serving as arbiter.

### Study characteristics

Data were extracted from all included studies by one reviewer. A random selection of 10% of the data was assessed by a second reviewer to determine the accuracy of the data extraction. A third reviewer served as arbiter in case of disagreements. In addition to bibliographical details (first author, year and journal), we extracted characteristics of the animal model used, including species, sex, age at the start of the study, comorbidities and whether the animals were immunocompromised. Details on all relevant experimental groups were extracted, including the number of animals in each group, the type of treatment and control(s) used, the timing of the intervention relative to HLI induction, the number of administrations, dose, administration route, administration site and timing of the outcome assessment. Regarding the cells and cell donors, we extracted data on the cell type, donor species, sex, age and comorbidities, whether the cells were allogenic to the recipient and whether cells had been cryopreserved before use.

For studies in which the dose was unclear, the number of cells reported was presumed to be the total dose given (Yao et al., 2020; Huang et al., 2014; Park et al., 2018; Sun et al., 2017; Tanaka et al., 2010). For one study mentioning animals being divided into groups without giving exact numbers (Li et al., 2021), an equal distribution was assumed.

### Risk of bias assessment

The risk of bias was assessed for each article according to the SYRCLE risk of bias tool and the determinants specified therein (Hooijmans et al., 2014) by two independent reviewers. Briefly, the risk of selection, performance, detection, attrition and reporting bias was assessed as being low, unclear or high. In addition to the risk of bias, the SYRCLE tool includes reporting of key quality indicators (yes versus no), which we assessed. The key quality indicators report (1) any randomization, (2) any masking, (3) a sample size calculation, (4) preregistration of a study protocol and (5) a conflict-of-interest statement. Disagreements were resolved through discussion or, if consensus could not be reached, by a third reviewer serving as arbiter. Both assessments focused on the experimental groups and outcome relevant for this evidence synthesis.

### Outcome data extraction

The relative perfusion in each experimental group was extracted in percentages. The number of animals in each group, the mean and the standard deviation (s.d.) were recorded. Where necessary, the s.d. was recalculated from the standard error of the mean (s.e.m.). If no numerical data were reported, data were extracted from graphs using FIJI. If the number of animals in a group was given as a range, the lowest number was used as a conservative estimate. If multiple cell types were assessed, outcome data from each cell type were included separately.

If the outcome was measured at multiple time points, data from the time point of highest efficacy (maximal difference between the control and treatment group) were recorded for the main analysis to study the maximal efficacy of treatment. Data from the latest available time point were recorded for the sensitivity analysis (see Results).

### Meta-analysis

The meta-analysis was performed in R using the ‘meta’ (Balduzzi et al., 2019) and ‘metafor’ (Viechtbauer, 2010) packages (see supplementary Materials and Methods for R scripts). The difference in relative perfusion between the treatment group and the control group was expressed as the mean difference and the corresponding 95% CI and pooled using a random-effects model. Between-study heterogeneity was assessed using I^2^ and R^2^.

To identify potential determinants of treatment efficacy (sources of heterogeneity), subgroup analyses were conducted based on animal species, comorbidities, whether the animals were immunocompromised, the administration route and dose, the cell type, donor species, whether the cells used were allogenic or from a diseased donor, and whether the cells were cryopreserved. All subgroup analyses were performed using stratified meta-regression, except for ‘dose’, for which a linear regression was used. In each subgroup analysis, we calculated the R^2^ statistic, which indicates the percentage of the heterogeneity that was accounted for by the variable, along with its *P*-value and the residual I^2^ statistic, which expresses the remaining variation due to between-study heterogeneity.

The main analyses were performed using the data extracted at the time point of maximum efficacy. To assess the robustness of this approach, a sensitivity analysis was performed using the data from the latest available time point.

As there were ten subgrouping variables, we used the Bonferroni–Holmes correction to determine the level of significance at *P*=0.005 instead of *P*=0.05.

### Publication bias

Publication bias was assessed through visual inspection of a funnel plot and a statistical assessment of asymmetry using trim-and-fill analysis (Viechtbauer, 2010).

## Supplementary Material

10.1242/dmm.050632_sup1 Supplementary information

Table S1. Full text exclusion reasons table

Table S3. Extended study characteristics

Table S4. Extended risk of bias analysis data
