# Supplementary material for: Systematic review and meta-analysis of the effect of bone marrow-derived cell therapies on hind limb perfusion
Source: Dis Model Mech. 2024 May 24;17(5):dmm050632. doi: 10.1242/dmm.050632 (PMC11139036; doi:10.1242/dmm.050632)
Supplement: Supplementary information [file dmm-17-050632-s1.pdf]

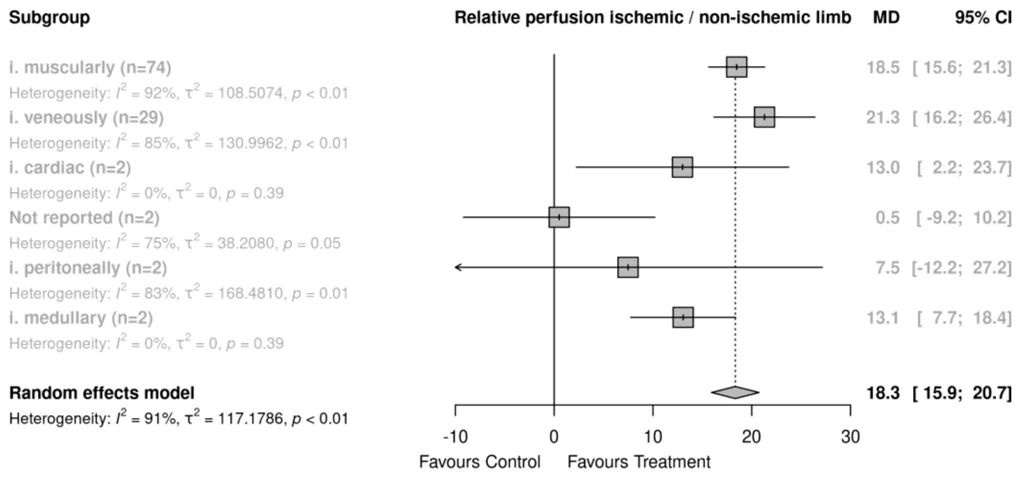

Fig. S1. Forest plot for administration route

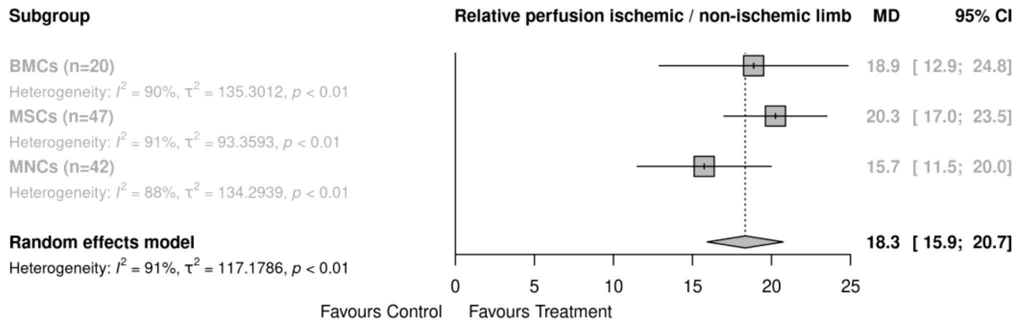

Fig. S2. Forest plot for cell type

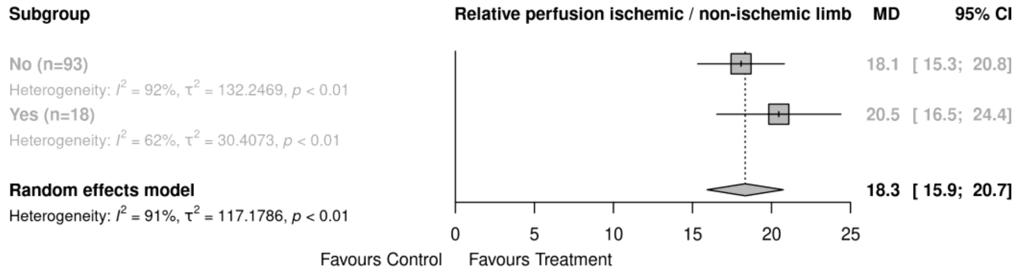

Fig. S3. Forest plot for comorbidities

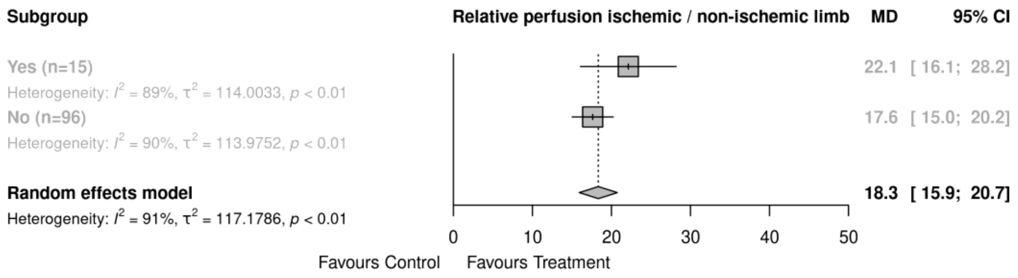

Fig. S4. Forest plot for cryopreservation

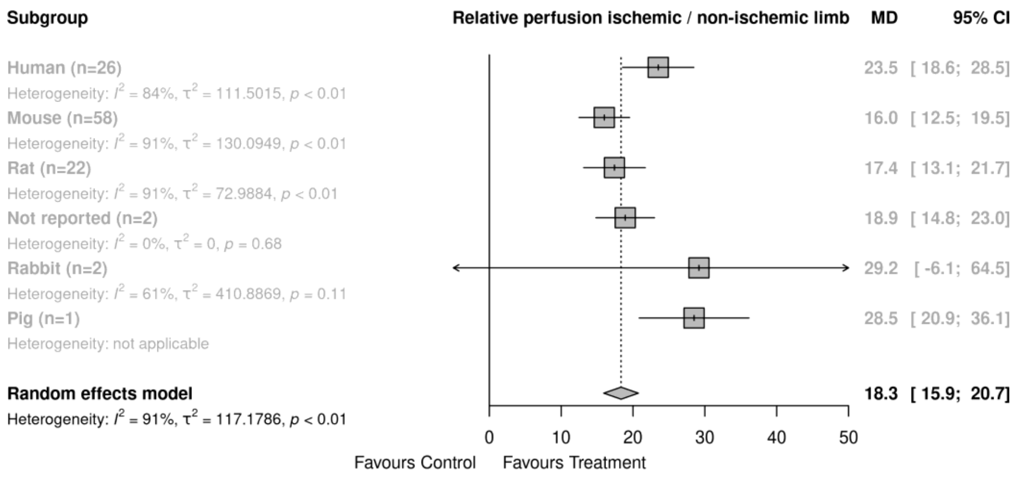

Fig. S5. Forest plot for donor species

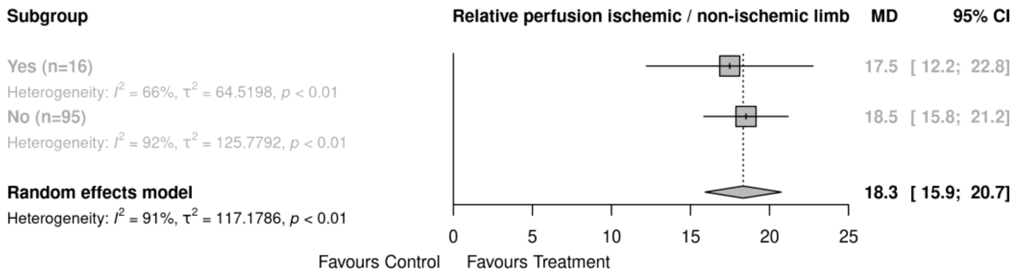

Fig. S6. Forest plot for donor comorbidity

Disease Models & Mechanisms • Supplementary information

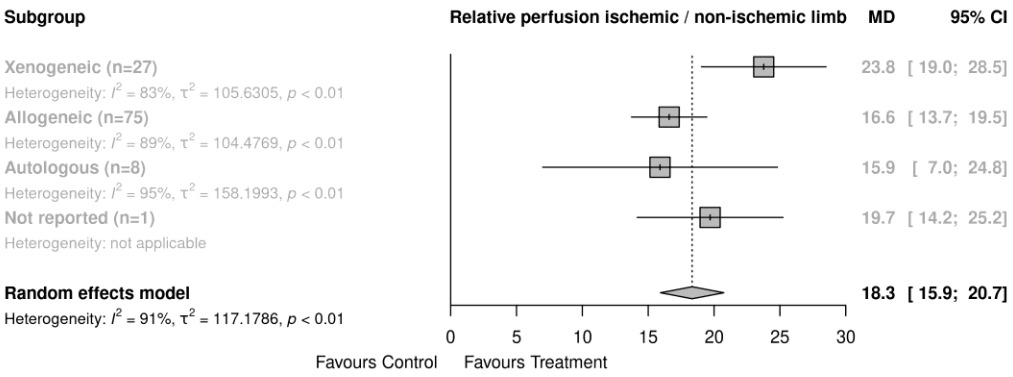

Fig. S7. Forest plot for genotype (cell origin)

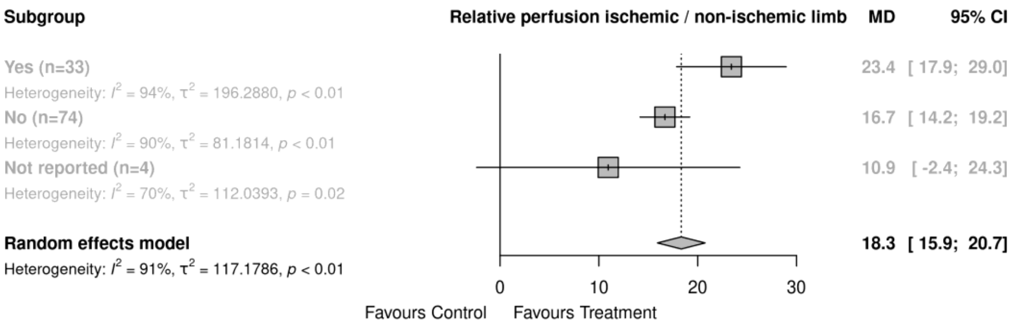

Fig. S8. Forest plot for immunostatus (immunocompromised Y/N)

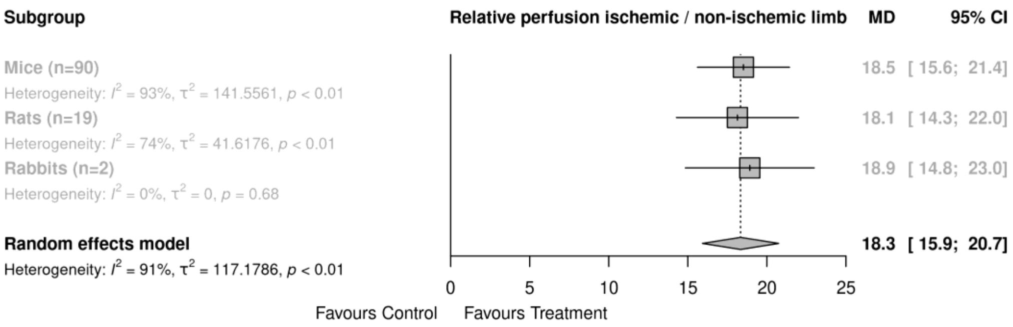

Fig. S9. Forest plot for species used.

Disease Models & Mechanisms • Supplementary information

Table S1. Full text exclusion reasons table

Available for download at
https://journals.biologists.com/dmm/article-lookup/doi/10.1242/dmm.050632#supplementary-data

Table S2. HLI induction methods

HLI induction method	Article count
Electrocautery and excision	1
Electrocoagulation	1
Ligation	20
Ligation and electrocoagulation	6
Ligation and excision	44
Ligation and transection	5
ligation, cautery and excision	1
Unclear	3
Not recorded	4
	Total
	85

Table S3. Extended study characteristics

Available for download at
https://journals.biologists.com/dmm/article-lookup/doi/10.1242/dmm.050632#supplementary-data

Table S4. Extended risk of bias analysis data

Available for download at
https://journals.biologists.com/dmm/article-lookup/doi/10.1242/dmm.050632#supplementary-data

Disease Models & Mechanisms • Supplementary information

Table S5. Subgroup statistics, related to figures 3,4,5 and supplemental figures of forest plots.

**Time point maximum effect**	**# comp**	**MD**	**95% CI**	**I^2**		
Overall	111	18.3	[15.9; 20.7]	92%		
				**Res. I^2**	**R^2**	**p-value (0.005 = sig)**
Species				92%	0%	0.98
Mice	90					
Rats	19					
Rabbits	2					
Recipient co-morbidity				92%	0%	0.5
No	93					
Yes	18					
Recipient Immunocompromised				92%	4%	0.03
Yes						
No						
Not reported						
Administration route				92%	6%	0.1
Intra muscularly	74			92%		
Intra veneously	29			85%		
Intra cardiac	2			0%		
Not reported	2			75%		
Intra peritoneally	2			83%		
Intra medullary	2			0%		
Cell dose (linear)	110	-	-	92%	0%	0.7
Donor cell type				92%	2%	0.2
BMCs	20					
MSCs	49					
MNCs	42					
Donor cell species				92%	7%	0.1
Human	26			84%		
Mouse	58			91%		
Rat	22			91%		
Not reported	2			0%		
Rabbit	2			61%		
Pig	1			NA		
Donor genotype				92%	6%	0.08
Xenogeneic	27			83%		
Allogeneic	75			89%		
Autogeneic	8			95%		
Not reported	1			NA		

Disease Models & Mechanisms • Supplementary information

Donor co-morbidity				92%	0%	0.7
Yes	16					
No	95					
Cell cryopreserved				92%	3%	0.2
Yes	15					
No	96					

Comp = experimental comparisons; MD = mean difference; CI = confidence interval. The p-value provided is from the test for moderators in meta-regression

MaxPerfusiontrimfill # show the analysis results
Number of studies: k = 147 (with 36 added studies)

95%-CI z p-value
Random effects model 11.4048 [8.5438; 14.2658] 7.81 < 0.0001

Quantifying heterogeneity:
tau^2 = 247.3653 [193.3523; 351.5210]; tau = 15.7278 [13.9051; 18.7489]
I^2 = 93.9% [93.2%; 94.5%]; H = 4.05 [3.85; 4.27]

Linear regression test of funnel plot asymmetry

Test result: t = 3.25, df = 109, p-value = 0.0015

Sample estimates:
bias se.bias intercept se.intercept
1.4592 0.4488 10.8313 1.3638

**Sensitivity analysis Latest Time Point**	**# comp**	**MD**	**95% CI**	**I^2**
Overall	111	17.6	[14.9; 20.2]	92%
	**Res. I^2**	**R^2**	**p-value (0.005 = sig)**	
Species	93%	5%	0.2	
Recipient co-morbidity	94%	0%	0.4	
Recipient Immunocompromised	93%	3%	0.08	
Administration route	93%	12%	0.01	
Cell dose (linear)	94%	0%	0.95	
Donor cell type	93%	0%	0.4	
Donor cell species	93%	5%	0.2	
Donor genotype	94%	0%	0.9	
Donor co-morbidity	94%	0%	0.4	
Cell cryopreserved	93%	2%	0.3	

Comp = experimental comparisons; MD = mean difference; CI = confidence interval. The p-value provided is from the test for moderators in meta-regression

Disease Models & Mechanisms • Supplementary information

Supplementary Materials and Methods

Complete search terms

EMBASE	18012021	1579 hits
#1	Ischemia	ischemia/ or experimental ischemia/ or exp peripheral ischemia/ or exp cold ischemia/ or exp muscle ischemia/ OR (ischemia OR ischaemia OR ischemic OR ischaemic OR ischemically OR ischaemically OR postischemic OR postischaemic OR post-ischemic OR post-ischaemic).ti,ab.
#2	Limb	exp limb/ OR (extremity OR extremities OR limb OR limbs OR hindlimb OR hindlimbs OR forelimb OR forelimbs OR leg OR legs OR foot OR feet OR hindleg OR hindlegs OR foreleg OR forelegs OR arm OR arms OR paw OR paws OR hindpaw OR hindpaws OR forepaw OR forepaws).ti,ab.
#3	Limb AND ischemia	(#1 AND #2)
#4	PAOD	exp limb ischemia/ OR exp critical limb ischemia/ OR exp leg ischemia/ OR peripheral occlusive artery disease/ OR blood vessel occlusion/ OR exp Buerger disease/ OR limb blood flow/ OR (femoral artery ligation OR femoral artery primary ligation OR femoral arterial ligation OR iliac artery ligation OR iliac arterial ligation OR peripheral arterial disease OR peripheral arterial diseases OR peripheral artery disease OR peripheral artery diseases OR peripheral arterial occlusi* OR peripheral artery occlusi* OR peripheral arterial obliterati* OR peripheral artery obliterati* OR peripheral arterial obstructi* OR peripheral artery obstructi* OR PAOD OR peripheral occlusive arterial disease OR peripheral occlusive arterial diseases OR peripheral obstructive arterial disease OR peripheral obstructive arterial diseases OR peripheral obliterative arterial disease OR peripheral obliterative arterial diseases OR peripheral occlusive artery disease OR peripheral occlusive artery diseases OR peripheral obstructive artery disease OR peripheral obstructive artery diseases OR peripheral obliterative artery disease OR peripheral obliterative artery diseases OR POAD OR arteriosclerosis obliterans OR atherosclerosis obliterans OR peripheral arteriosclerotic occlusion OR peripheral atherosclerotic occlusion OR peripheral arteriolerotic occlusions OR peripheral atherosclerotic occlusions OR peripheral arterial insufficiency OR peripheral arterial insufficiencies OR peripheral artery insufficiency OR peripheral artery insufficiencies OR thromboangitis obliterans OR Buerger* OR ischemic ulcer OR ischaemic ulcer OR ischemic ulcers OR ischaemic ulcers OR gangrene).ti,ab.
#5	(limb AND ischemia) OR PAOD	#3 OR #4
#6	stem cells	exp stem cell/ OR exp stem cell research/ OR exp stem cell transplantation/ OR exp bone marrow cell/ OR (stem cell OR stem cells OR stromal cell OR stromal cells OR progenitor cell OR progenitor cells OR precursor cell OR precursor cells OR mother cell OR mother cells OR cell therapy OR cell therapies OR cell-based therapy OR cellbased therapy OR cell-based therapies OR cellbased therapies OR cell-based treatment OR cellbased treatment OR cell-based treatments OR cellbased treatments OR MSC).ti,ab. OR ((bone marrow OR bonemarrow OR omnipotent OR pluripotent).ti,ab. AND (cell OR cells).ti,ab.)
#7	animals	Laboratory animal search filter [2]
#8	limb ischemia OR PAOD AND stem cells AND animals	#5 AND #6 AND #7
	limit 8 to (article or article in press or conference paper)	

Disease Models & Mechanisms • Supplementary information

PubMed	04012021	1508 hits
#1	Ischemia	ischemia[Mesh] OR "warm ischemia"[Mesh] OR “cold ischemia”[Mesh] OR ischemia[tiab] OR ischaemia[tiab] OR ischemic[tiab] OR ischaemic[tiab] OR ischemically[tiab] OR ischaemically[tiab] OR postischemic[tiab] OR postischaemic[tiab] OR “post-ischemic”[tiab] OR “post-ischaemic”[tiab]
#2	Limb	extremities[Mesh] OR hindlimb[Mesh] OR extremity[tiab] OR extremities[tiab] OR limb[tiab] OR limbs[tiab] OR hindlimb[tiab] OR hindlimbs[tiab] OR forelimb[tiab] OR forelimbs[tiab] OR leg[tiab] OR legs[tiab] OR foot[tiab] OR feet[tiab] OR hindleg[tiab] OR hindlegs[tiab] OR foreleg[tiab] OR forelegs[tiab] OR arm[tiab] OR arms[tiab] OR paw[tiab] OR paws[tiab] OR hindpaw[tiab] OR hindpaws[tiab] OR forepaw[tiab] OR forepaws[tiab]
#3	Limb AND ischemia	(#1 AND #2)
#4	PAOD	"Peripheral Arterial Occlusive Disease 1" [Supplementary Concept] OR "Thromboangiitis Obliterans"[Mesh] OR “femoral artery ligation”[tiab] OR “femoral artery primary ligation”[tiab] OR “femoral arterial ligation”[tiab] OR “iliac artery ligation”[tiab] OR “iliac arterial ligation”[tiab] OR “peripheral arterial disease”[tiab] OR “peripheral arterial diseases”[tiab] OR “peripheral artery disease”[tiab] OR “peripheral artery diseases”[tiab] OR “peripheral arterial occlusi*”[tiab] OR “peripheral artery occlusi*”[tiab] OR “peripheral arterial obliterati*”[tiab] OR “peripheral artery obliterati*”[tiab] OR “peripheral arterial obstructi*”[tiab] OR “peripheral artery obstructi*”[tiab] OR PAOD[tiab] OR “peripheral occlusive arterial disease”[tiab] OR “peripheral occlusive arterial diseases”[tiab] OR “peripheral obstructive arterial disease”[tiab] OR “peripheral obstructive arterial diseases”[tiab] OR “peripheral obliterative arterial disease”[tiab] OR “peripheral obliterative arterial diseases”[tiab] OR “peripheral occlusive artery disease”[tiab] OR “peripheral occlusive artery diseases”[tiab] OR “peripheral obstructive artery disease”[tiab] OR “peripheral obstructive artery diseases”[tiab] OR “peripheral obliterative artery disease”[tiab] OR “peripheral obliterative artery diseases”[tiab] OR POAD[tiab] OR “arteriosclerosis obliterans”[tiab] OR “atherosclerosis obliterans”[tiab] OR “peripheral arteriosclerotic occlusion”[tiab] OR “peripheral atherosclerotic occlusion”[tiab] OR “peripheral arteriolerotic occlusions”[tiab] OR “peripheral atherosclerotic occlusions”[tiab] OR “peripheral arterial insufficiency”[tiab] OR “peripheral arterial insufficiencies”[tiab] OR “peripheral artery insufficiency”[tiab] OR “peripheral artery insufficiencies”[tiab] OR “thromboangitis obliterans”[tiab] OR Buerger*[tiab] OR “ischemic ulcer”[tiab] OR “ischaemic ulcer”[tiab] OR “ischemic ulcers”[tiab] OR “ischaemic ulcers”[tiab] OR gangrene[tiab]
#5	(limb AND ischemia) OR PAOD	#3 OR #4
#6	stem cells	("stem cells"[MeSH] OR "Stem Cell Research"[Mesh] OR "Stem Cell Transplantation"[Mesh] OR "Bone Marrow Cells"[Mesh] OR "stem cell"[tiab] OR "stem cells"[tiab] OR "stromal cell"[tiab] OR "stromal cells"[tiab] OR "progenitor cell"[tiab] OR "progenitor cells"[tiab] OR “precursor cell”[tiab] OR “precursor cells”[tiab] OR "mother cell"[tiab] OR "mother cells"[tiab] OR "cell therapy"[tiab] OR "cell therapies"[tiab] OR "cell-based therapy"[tiab] OR "cellbased therapy"[tiab] OR "cell-based therapies"[tiab] OR "cellbased therapies"[tiab] OR "cell-based treatment"[tiab] OR "cellbased treatment"[tiab] OR "cell-based treatments"[tiab] OR "cellbased treatments"[tiab] OR MSC[tiab]) OR (("bone marrow"[tiab] OR bonemarrow[tiab] OR omnipotent[tiab] OR pluripotent[tiab]) AND (cell[tiab] OR cells[tiab]))
#7	animals	Laboratory animal search filter [1]
#8	limb ischemia OR PAOD AND stem cells AND animals	#5 AND #6 AND #7 NOT “review”[ptyp]

Code used for meta-analysis

```
#Needed packages:
install.packages("statmod")
install.packages("metafor")
install.packages("meta")
install.packages("lifecycle")
install.packages("readr")
install.packages("tidyverse")

#Load
library(tidyverse)
library(statmod)
library(readr)
library(lifecycle)
library(meta)
library(metafor)

####Set Up and Load##########
#set working directory to USB drive
setwd("F:/Biology Radboud/#Internship SYRCLE/R analysis")

#Load Data
MaxPerfusion <- read_csv("data/MPRAnalysis.csv")
View(MaxPerfusion)

LastPerfusion <- read_csv("data/LDRAnalysis.csv")
View(LastPerfusion)

#######Analysis Max Perfusion##########
#Max Perfusion meta- analysis
MaxPerfusionMA <- metagen(
  MD, MDSE,
  data = MaxPerfusion,
  studlab = Article_Code,
  random = TRUE,
  method.tau = "REML")

MaxPerfusionMA

#Overall forest plot
png(filename="outcomes/MaxPerfusion.png",width=750,height=950)
MaxPerfusionPlot <- forest(
  MaxPerfusionMA,
  sortvar = TE,
  fixed = FALSE,
  random = TRUE,
  leftlabs = c("Study", "ES", "SE"),
  xlab = "Perfusion ischemic/non ischemic leg (%)",
  smlab = "Effect Size Perfusion",
  col.square = "darkseagreen1", col.inside = "black",
```

Disease Models & Mechanisms • Supplementary information

```
  label.right = "                Favours Treatment",
  label.left = "Favours Control",
  xlim = c(-40,100),
  fontsize = 10,
  plotwidth = "10cm",
  digits = 2,
  digits.se = 2 )
dev.off()

############SPECIES###########
#Subgroup Species Statistics
AnalysisSpecies <- metareg(
  MaxPerfusionMA, ~ Species)

AnalysisSpecies

#Species Graph
MaxPerfusionSpecies <- update(MaxPerfusionMA, subgroup = Species)

png(filename="outcomes/Species.png",width=750,height=1100)
SpeciesPlot <- forest(
  MaxPerfusionSpecies,
  sortvar = TE,
  fixed = FALSE,
  random = TRUE,
  leftlabs = c("Study", "ES", "SE"),
  xlab = "Perfusion ischemic/non ischemic leg (%)",
  smlab = "Effect Size Perfusion",
  col.square = "darkseagreen1", col.inside = "black", col.by = "grey30",
  label.right = "                Favours Treatment",
  label.left = "Favours Control",
  xlim = c(-40,100),
  fontsize = 10,
  plotwidth = "10cm",
  digits = 2,
  digits.se = 2 )
dev.off()

############COMORBIDITIES################
#Subgroup Comorbidities Statistics
AnalysisComorbidities <- metareg(
  MaxPerfusionMA, ~ Comorbidities)

AnalysisComorbidities

#Comorbidities Graph
MaxPerfusionComorbidities <- update(MaxPerfusionMA, subgroup = Comorbidities)

png(filename="outcomes/Comobidities.png",width=750,height=1300)
ComorbiditiesPlot <- forest(
  MaxPerfusionComorbidities,
```

Disease Models & Mechanisms • Supplementary information

```
  sortvar = TE,
  fixed = FALSE,
  random = TRUE,
  leftlabs = c("Study", "ES", "SE"),
  xlab = "Perfusion ischemic/non ischemic leg (%)",
  smlab = "Effect Size Perfusion",
  col.square = "darkseagreen1", col.inside = "black", col.by = "grey30",
  label.right = "                Favours Treatment",
  label.left = "Favours Control",
  xlim = c(-40,100),
  fontsize = 10,
  plotwidth = "10cm",
  digits = 2,
  digits.se = 2 )
dev.off()

############IMMUNO#############
#Subgroup Immuno Statistics
AnalysisImmuno <- metareg(
  MaxPerfusionMA, ~ Immunocomprimised)

AnalysisImmuno

# Immuno Graph
MaxPerfusionImmuno <- update(MaxPerfusionMA, subgroup = Immunocomprimised)

png(filename="outcomes/Immuno.png",width=750,height=1100)
ImmunoPlot <- forest(
  MaxPerfusionImmuno,
  sortvar = TE,
  fixed = FALSE,
  random = TRUE,
  leftlabs = c("Study", "ES", "SE"),
  xlab = "Perfusion ischemic/non ischemic leg (%)",
  smlab = "Effect Size Perfusion",
  col.square = "darkseagreen1", col.inside = "black", col.by = "grey30",
  label.right = "                Favours Treatment",
  label.left = "Favours Control",
  xlim = c(-40,100),
  fontsize = 10,
  plotwidth = "10cm",
  digits = 2,
  digits.se = 2 )
dev.off()

############ADMIN#############
#Subgroup Admin Statistics
AnalysisAdmin <- metareg(
  MaxPerfusionMA, ~ Admin_route)

AnalysisAdmin
```

Disease Models & Mechanisms • Supplementary information

```
# Admin Graph
MaxPerfusionAdmin <- update(MaxPerfusionMA, subgroup = Admin_route)

png(filename="outcomes/Admin route.png",width=750,height=1200)
AdminPlot <- forest(
  MaxPerfusionAdmin,
  sortvar = TE,
  fixed = FALSE,
  random = TRUE,
  leftlabs = c("Study", "ES", "SE"),
  xlab = "Perfusion ischemic/non ischemic leg (%)",
  smlab = "Effect Size Perfusion",
  col.square = "darkseagreen1", col.inside = "black", col.by = "grey30",
  label.right = "                Favours Treatment",
  label.left = "Favours Control",
  xlim = c(-40,100),
  fontsize = 10,
  plotwidth = "10cm",
  digits = 2,
  digits.se = 2 )
dev.off()

############DOSE#############
#Subgroup dose Statistics
AnalysisDose <- metareg(
  MaxPerfusionMA, ~ Dose_full)

AnalysisDose

# Dose Graph
png(filename="outcomes/Dose Regression.png",width=850,height=500)
bubble(AnalysisDose,
       ylim = c(-20,80),
       xlab = "Dose (# of cells)",
       ylab = "Effect size",
       lwd = 1,
       col = "darkblue",
       bg = "transparent",
       col.line = "darkred",
       min.cex = 0.5,
       max.cex = 3,
       las = 1,
       cex.axis = 1.5,
       cex.lab = 1.5)
dev.off()

############CELL TYPE#############
#Subgroup Cell Type Statistics
AnalysisCells <- metareg(
  MaxPerfusionMA, ~ Cells)
```

Disease Models & Mechanisms • Supplementary information

```
AnalysisCells

#Cell Graph
MaxPerfusionCells <- update(MaxPerfusionMA, subgroup = Cells)

png(filename="outcomes/Cells.png",width=750,height=1100)
CellTypePlot <- forest(
  MaxPerfusionCells,
  sortvar = TE,
  fixed = FALSE,
  random = TRUE,
  leftlabs = c("Study", "ES", "SE"),
  xlab = "Perfusion ischemic/non ischemic leg (%)",
  smlab = "Effect Size Perfusion",  #
  col.square = "darkseagreen1", col.inside = "black", col.by = "grey30",
  label.right = "                Favours Treatment",
  label.left = "Favours Control",
  xlim = c(-40,100),
  fontsize = 10,
  plotwidth = "10cm",
  digits = 2,
  digits.se = 2 )
dev.off()

############DONOR SPECIES#############
#Subgroup Donor Species Statistics
AnalysisDonorSpecies <- metareg(
  MaxPerfusionMA, ~ Donor_Species)

AnalysisDonorSpecies

#Donor Species Graph
MaxPerfusionDonorSpecies <- update(MaxPerfusionMA, subgroup = Donor_Species)

png(filename="outcomes/Donor Species.png",width=750,height=1300)
DonorSpeciesPlot <- forest(
  MaxPerfusionDonorSpecies,
  sortvar = TE,
  fixed = FALSE,
  random = TRUE,
  leftlabs = c("Study", "ES", "SE"),
  xlab = "Perfusion ischemic/non ischemic leg (%)",
  smlab = "Effect Size Perfusion",
  col.square = "darkseagreen1", col.inside = "black", col.by = "grey30",
  label.right = "                Favours Treatment",
  label.left = "Favours Control",
  xlim = c(-40,100),
  fontsize = 10,
  plotwidth = "10cm",
  digits = 2,
```

Disease Models & Mechanisms • Supplementary information

```
  digits.se = 2 )
dev.off()

############ALLOGENIC#############
#Subgroup Allogenic Statistics
AnalysisAllogenic <- metareg(
  MaxPerfusionMA, ~ Allogenic)

AnalysisAllogenic

#Allogenic Graph
MaxPerfusionAllogenic <- update(MaxPerfusionMA, subgroup = Allogenic)

png(filename="outcomes/Allogenic.png",width=750,height=1100)
AllogenicPlot <- forest(
  MaxPerfusionAllogenic,
  sortvar = TE,
  fixed = FALSE,
  random = TRUE,
  leftlabs = c("Study", "ES", "SE"),
  xlab = "Perfusion ischemic/non ischemic leg (%)",
  smlab = "Effect Size Perfusion",
  col.square = "darkseagreen1", col.inside = "black", col.by = "grey30",
  label.right = "                Favours Treatment",
  label.left = "Favours Control",
  xlim = c(-40,100),
  fontsize = 10,
  plotwidth = "10cm",
  digits = 2,
  digits.se = 2 )
dev.off()

############DISEASED DONOR#############
#Subgroup Diseased Donor Statistics
AnalysisDiseasedDonor <- metareg(
  MaxPerfusionMA, ~ Diseased_Donor)

AnalysisDiseasedDonor

#Diseased Donor Graph
MaxPerfusionDiseasedDonor <- update(MaxPerfusionMA, subgroup = Diseased_Donor)

png(filename="outcomes/Diseased Donor.png",width=750,height=1200)
DiseasedDonorPlot <- forest(
  MaxPerfusionDiseasedDonor,
  sortvar = TE,
  fixed = FALSE,
  random = TRUE,
  leftlabs = c("Study", "ES", "SE"),
  xlab = "Perfusion ischemic/non ischemic leg (%)",
  smlab = "Effect Size Perfusion",
```

Disease Models & Mechanisms • Supplementary information

```
  col.square = "darkseagreen1", col.inside = "black", col.by = "grey30",
  label.right = "                Favours Treatment",
  label.left = "Favours Control",
  xlim = c(-40,100),
  fontsize = 10,
  plotwidth = "10cm",
  digits = 2,
  digits.se = 2 )
dev.off()

############CRYOPRESERVATION#############
#Subgroup Cryopreservation Statistics
AnalysisCryo <- metareg(
  MaxPerfusionMA, ~ Cryopreservation)

AnalysisCryo

#Diseased Donor Graph
MaxPerfusionCryo <- update(MaxPerfusionMA, subgroup = Cryopreservation)

png(filename="outcomes/Cryopreservation.png",width=750,height=1100)
CryoPlot <- forest(
  MaxPerfusionCryo,
  sortvar = TE,
  fixed = FALSE,
  random = TRUE,
  leftlabs = c("Study", "ES", "SE"),
  xlab = "Perfusion ischemic/non ischemic leg (%)",
  smlab = "Effect Size Perfusion",
  col.square = "darkseagreen1", col.inside = "black", col.by = "grey30",
  label.right = "                Favours Treatment",
  label.left = "Favours Control",
  xlim = c(-40,100),
  fontsize = 10,
  plotwidth = "10cm",
  digits = 2,
  digits.se = 2 )
dev.off()

######Day sensitivity test############
LastDayPerfusionMA <- metagen(
  MD, MDSE,
  data = LastPerfusion,
  studlab = Article_Code,
  random = TRUE,
  method.tau = "REML")

LastDayPerfusionMA

#Graph
png(filename="outcomes/LastDay.png",width=750,height=950)
```

Disease Models & Mechanisms • Supplementary information

```
LastDayPlot <- forest(
  LastDayPerfusionMA,
  sortvar = TE,
  fixed = FALSE,
  random = TRUE,
  leftlabs = c("Study", "ES", "SE"),
  xlab = "Perfusion ischemic/non ischemic leg (%)",
  smlab = "Effect Size Perfusion",
  col.square = "darkseagreen1", col.inside = "black",
  label.right = "                Favours Treatment",
  label.left = "Favours Control",
  xlim = c(-40,100),
  fontsize = 10,
  plotwidth = "10cm",
  digits = 2,
  digits.se = 2 )
dev.off()

##########Publication Bias###############
#funnel plot
png(filename="outcomes/Funnel.png",width=600,height=550)
MaxPerfusionMAFunnel <- funnel(MaxPerfusionMA, xlab = "Effect size",
                               bg=3,
                               las = 1,
                               cex.axis = 1.5,
                               cex.lab = 1.5,
                               cex = 1.4)
dev.off()

#Trim and fill
TrimMP <- trimfill(MaxPerfusionMA, random = TRUE, method.tau = "REML")

png(filename="outcomes/Funnel Trim.png",width=600,height=550)
MaxPerfusionMAFunnelTrim <- funnel(TrimMP, xlab = "Effect size",
                                   bg=3,
                                   las = 1,
                                   cex.axis = 1.5,
                                   cex.lab = 1.5,
                                   cex = 1.4)
abline(v = 20.7702, lty = 3)
abline(v = 0, lty = 3, col = "gray80")
dev.off()

#Funnel plot no triangle
png(filename="outcomes/Funnel Scatter.png",width=600,height=550)
plot(MaxPerfusionMA$TE, MaxPerfusionMA$seTE, xlim = c(-20,80),
     ylim = rev(range(MaxPerfusionMA$seTE)),
     xlab = "Effect size",
     ylab = "Standard Error",
     cex = 1.4,
     pch= 21,
```

```
    bg = 3)
abline(v = 20.7702, lty = 3)
abline(v = 0, lty = 3, col = "gray80")
dev.off()
```

Disease Models & Mechanisms • Supplementary information
